# Hydnocarpin, a Natural Flavonolignan, Induces the ROS-Mediated Apoptosis of Ovarian Cancer Cells and Reprograms Tumor-Associated Immune Cells

**DOI:** 10.3390/antiox14070846

**Published:** 2025-07-10

**Authors:** Jae-Yoon Kim, Yejin Kim, Soo-Yeon Woo, Jin-Ok Kim, Hyunsoo Kim, So-Ri Son, Dae Sik Jang, Jung-Hye Choi

**Affiliations:** 1 Department of Biomedical and Pharmaceutical Sciences, Kyung Hee University, Seoul 02447, Republic of Korea; yoonie1028@khu.ac.kr (J.-Y.K.); yezeen@khu.ac.kr (Y.K.); sooyeon_woo@khu.ac.kr (S.-Y.W.); nayaajo@khu.ac.kr (J.-O.K.); suhanyak@hanbanglife.com (H.K.); dsjang@khu.ac.kr (D.S.J.); 2 College of Pharmacy, Kyung Hee University, Seoul 02447, Republic of Korea; allosori@khu.ac.kr

**Keywords:** ovarian cancer, tumor microenvironment, *Pueraria Flos*, hydnocarpin, reactive oxidative stress, tumor-associated macrophages, T cells

## Abstract

Ovarian cancer, the most lethal form of gynecological cancer worldwide with a poor prognosis, is largely driven by an immunosuppressive tumor microenvironment. In this study, we investigated the anticancer effects of hydnocarpin, a natural flavonolignan derived from the flowers of *Pueraria lobata*, focusing on its effects on ovarian cancer and tumor-associated immune cells, including ovarian cancer-stimulated macrophages (MQs) and T cells. Hydnocarpin exhibited potent cytotoxicity against multiple ovarian cancer cell lines but only minimal toxicity against normal ovarian surface epithelial cells. Mechanistically, hydnocarpin triggered caspase-dependent apoptosis, as evidenced by the activation of caspase-9 and -3, with limited involvement of caspase-8, indicating the activation of the intrinsic apoptotic pathway. Experimental data implicated reactive oxygen species generation as a key mediator of hydnocarpin cytotoxicity, and reactive oxygen species inhibition significantly inhibited this cytotoxicity. In addition to its direct tumoricidal effects, hydnocarpin reprogrammed the tumor-associated immune cells, ovarian cancer-stimulated macrophages and T cells, by downregulating the levels of M2 MQ markers and pro-tumoral factors (matrix metalloproteinase-2/9, C–C motif chemokine ligand 5, transforming growth factor-β, and vascular endothelial growth factor) and enhancing MQ phagocytosis. Additionally, hydnocarpin promoted T-cell activation (interferon-γ and interleukin-2) and reduced the expression levels of immune evasion markers (CD80, CD86, and VISTA). Overall, this study demonstrated the dual anti-tumor effects of hydnocarpin on both ovarian cancer cells and immunosuppressive immune components in the tumor microenvironment, highlighting its potential as a novel therapeutic candidate for ovarian cancer.

## 1. Introduction

Ovarian cancer is the deadliest and third most frequently diagnosed cancer among all gynecologic malignancies worldwide [1,2]. Although initial treatment strategies—such as optimal cytoreductive surgery followed by platinum–taxane-based chemotherapy—can induce remission in many patients with advanced ovarian cancer, approximately 70–80% eventually experience disease recurrence, often within 18 months of initial treatment [3]. Recurrent ovarian cancer is frequently associated with the development of platinum resistance, which markedly reduces the effectiveness of standard therapies and severely limits subsequent treatment options [4]. While targeted agents such as poly (ADP-ribose) polymerase (PARP) inhibitors have improved outcomes, their clinical benefit is largely confined to patients with BRCA mutations or homologous recombination deficiency (HRD) [5]. Furthermore, immune checkpoint inhibitors (ICIs), including anti-PD-1/PD-L1 antibodies, have shown limited efficacy in ovarian cancer, partly due to its immunologically “cold” tumor microenvironment characterized by low T-cell infiltration and the presence of immunosuppressive cells [6]. The tumor microenvironment (TME) consists of various cells around the tumor, including immune cells such as macrophages (MQs) and T cells. The TME plays a crucial role in ovarian cancer development and is the main cause of poor patient prognosis [7]. Therefore, targeting both ovarian cancer cells and their microenvironment is a promising strategy for ovarian cancer treatment.

*Pueraria lobata*, native to the Pacific Islands and East and Southeast Asia, is a well-known medicinal herb containing diverse bioactive components [8,9]. The extract of its flowers, commonly known as *Pueraria Flos* (PF), and secondary metabolites exhibit various pharmacological activities, including anti-inflammatory [10,11], anti-diabetic [12], and anti-endometriotic [13] activities. Among its components, hydnocarpin, a flavone-type flavonolignan [14], has garnered attention owing to its multifaceted biological activities, including anti-biofilm activity against *Staphylococcus aureus* [15] and anti-inflammatory [16] and anti-acute lung injury [17] activities. Additionally, hydnocarpin exerts anti-tumor effects against various cancer types, including leukemia, colon cancer, and breast cancer [16,18,19,20,21,22]. To date, most studies have primarily focused on its direct cytotoxic effects, with a limited exploration of its molecular action mechanisms and immunomodulatory effects. We previously identified hydnocarpin as the most potent inhibitor of ovarian cancer cell growth among 22 PF constituents [14]. However, its precise anticancer mechanisms against ovarian cancer, particularly its roles in tumor-associated immune modulation, remain unclear. Therefore, in this study, we aimed to elucidate the molecular mechanisms underlying the cytotoxic effects of hydnocarpin on ovarian cancer cells and assess its immunomodulatory effects on tumor-associated MQs and T cells.

## 2. Materials and Methods

### 2.1. Sample Preparation

The authentication of the herbal material was carried out by Professor Dae Sik Jang, and a voucher specimen (PULO5-2019) was deposited in the professor’s laboratory at Kyung Hee University in Seoul, Republic of Korea. Preparation of the plant material and isolation of the compounds adhered to previously documented methods [14]. Briefly, the dried *Pueraria Flos* (1.6 kg) was extracted twice with methanol (10× *v*/*w*). The resulting extract was filtered and concentrated to obtain a crude extract (278.95 g). Subsequently, the crude extract was subjected to Diaion HP-20 column chromatography (CC; acetone/water = 0:100 to 100:0, *v*/*v*). An enriched fraction of (-)-hydnocarpin was further separated through a series of chromatographic procedures, employing Sephadex LH-20 (75% methanol) and silica gel CC (dichloromethane/methanol/water = 85:13.5:1.5 to 75:22.5:2.5, *v*/*v*/*v*), to yield a purified (-)-hydnocarpin (with purity > 95%).

### 2.2. Material

Roswell Park Memorial Institute (RPMI) 1640 and Dulbecco’s Modified Eagle Medium (DMEM) were procured from WELGENE Inc. (Gyeongsan, Republic of Korea). Penicillin–streptomycin was also obtained from WELGENE, while fetal bovine serum (FBS) was sourced from HyClone (Logan, UT, USA). Additional reagents utilized in this study included N-acetyl cysteine (NAC), phorbol myristate acetate (PMA), β-mercaptoethanol, and ionomycin calcium salt, all purchased from Sigma Chemical (St. Louis, MO, USA). The caspase inhibitor z-VAD-fmk was purchased from Calbiochem (Bad Soden, Germany). Thiazolyl Blue tetrazolium bromide (MTT) was sourced from Molecular Probes Inc. (Eugene, OR, USA). Antibodies against caspase-8 (51-80841N) and caspase-9 (9502S) were obtained from BD Pharmingen (Franklin Lakes, NJ, USA) and Cell Signaling (Beverly, MA, USA), respectively. Antibodies targeting caspase-3 (sc-7272) and GAPDH (sc-47724), 3,3-dihexyloxacarbocyanine iodide (DiOC_6_(3)), along with the 2′,7′-dichlorofluorescein diacetate (DCFH-DA) reagent, were acquired from Santa Cruz Biotechnology (Dallas, TX, USA).

### 2.3. Cell Culture

The human ovarian cancer cell lines A2780, SKOV3, ES2, and HeyA8, and the human monocyte cell line THP-1, were originally from the American Type Culture Collection (ATCC, Manassas, VA, USA). The human acute leukemia cell line CCRF-HSB2 was obtained from the Korean Cell Line Bank (Seoul, Republic of Korea). All cell lines (except CCRF-HSB2) were cultured in RPMI medium supplemented with 5% FBS (for A2780, SKOV3, ES2, and HeyA8) and 10% FBS (for THP-1). Penicillin–streptomycin was added to a final concentration of 1%. For THP-1 cells, 0.05 mM β-mercaptoethanol was also added. CCRF-HSB2 cells were maintained in DMEM with 10% FBS and 1% penicillin–streptomycin. These cells were activated with 100 nM PMA and 1nM ionomycin prior to experimental use. THP-1 cells were treated with 100 nM PMA for 24 h in order to induce differentiation into macrophages. Macrophages stimulated with ovarian cancer cells (OC-MQs) were generated by inducing THP-1-derived macrophages with the conditioned medium (CM) of A2780 cells. This method recapitulates tumor-induced macrophage polarization in vitro and is widely used to model TAM-like phenotypes in the ovarian cancer microenvironment.

### 2.4. Cell Viability Assay

A2780 and THP-1 cells were seeded and incubated 24 h before treatment. Subsequently, varying concentrations of hydnocarpin were added to the wells, followed by an additional 48 h incubation. MTT solution (0.5 mg/mL, 50 μL) was added to each well and incubated for 4 h. The formazan crystals were dissolved in DMSO, and absorbances were measured at 540 nm using a microplate reader (SpectraMax, Molecular Devices, Sunnyvale, CA, USA).

### 2.5. Annexin V/PI

Annexin V/PI staining was performed using the ApoScanTM kit (BioBud, Seoul, Republic of Korea) according to the manufacturer’s instructions. FITC-conjugated Annexin V and propidium iodide were analyzed using flow cytometry (CytoFlex, Beckman Coulter, Brea, CA, USA).

### 2.6. Western Blot

Cells were harvested and lysed using lysis buffer from Intron Biotechnology (Seoul, Republic of Korea). Equal amounts of proteins (30 μg) were separated by 15% SDSPAGE and transferred onto polyvinylidene difluoride (PDVF) membranes (Millipore, Bedford, MA, USA). Membranes were incubated with primary antibodies overnight at 4 °C, followed by incubation with horseradish peroxidase (HRP)-conjugated secondary antibody. Chemiluminescence signals were detected using reagents from Nicsro (Seoul, Republic of Korea) and imaged with the Image Quant LAS-4000 system from Fujifilm Life Science (Tokyo, Japan). Band intensities were normalized to the untreated control group and quantified using ImageJ software 1.51 (National Institutes of Health, Bethesda, MD, USA).

### 2.7. Mitochondrial Membrane Potential Assay

Mitochondrial membrane potential (Δψm) was assessed using DiOC_6_(3) [23,24]. A2780 cells were treated with hydnocarpin for the indicated time, harvested, and incubated with 50 nM DiOC_6_(3) for 30 min at 37 °C in the dark. Cells were then washed with PBS and analyzed by flow cytometry. A decrease in DiOC_6_(3) fluorescence intensity was interpreted as a loss of mitochondrial membrane potential.

### 2.8. DCFH-DA Assay

To detect intracellular reactive oxygen species (ROS), cells were treated with hydnocarpin for the indicated time. Cells were then harvested and stained with 100 μM DCFH-DA for 30 min in the dark. After washing with PBS, fluorescent intensity was measured by flow cytometry.

### 2.9. Reverse Transcriptase PCR

Total RNA was extracted using Easy Blue^®^ kits and reverse transcribed into cDNA. RT-PCR was performed with the SYBR Premix Ex Taq™ Kit (TakaRa, Kusatsu, Japan) using the CFX Connect Real-Time PCR Detection System (Bio-Rad Laboratories, Hercules, CA, USA). The comparative expression levels of the genes were analyzed using the 2−ΔΔCt method and compared with the expression level of β-actin. The primers utilized in this study are enumerated in Supplementary Table S1.

### 2.10. Phagocytosis Assay

For the confocal microscopy-based phagocytosis assay, 1.5 × 10^5^ THP-1 macrophages labeled with 7.5 µM CellTracker red (Thermofisher Scientific, Waltham, MA, USA) were seeded in a 4-well culture slide (SPL Life Sciences, Pocheon, Republic of Korea) and incubated for 24 h. The following day, 0.7 × 10^5^ A2780 cells stained with CellTracker green (Thermofisher scientific) and treated with hydnocarpin were added. After 48 h, cells were washed and fixed with methanol. Imaging was performed using a K1-Fluo confocal microscope (Daejeon, Republic of Korea) at ×200 magnification. Phagocytosis was quantified as the number of macrophages containing GFP+/RFP+ cells per 20 macrophages.

### 2.11. Statistical Analysis

All data are presented as mean ± standard deviation. Statistical analyses were performed using GraphPad Prism 8 software (San Diego, CA, USA). Student’s *t*-test was employed for comparisons between two groups, while one-way ANOVA was applied for multiple group comparisons. All experiments were conducted independently in triplicate, and a *p*-value of less than 0.05 was considered statistically significant.

## 3. Results

### 3.1. Hydnocarpin Induces the Caspase-Dependent Apoptosis of Human Ovarian Cancer Cells

**Figure 1:**
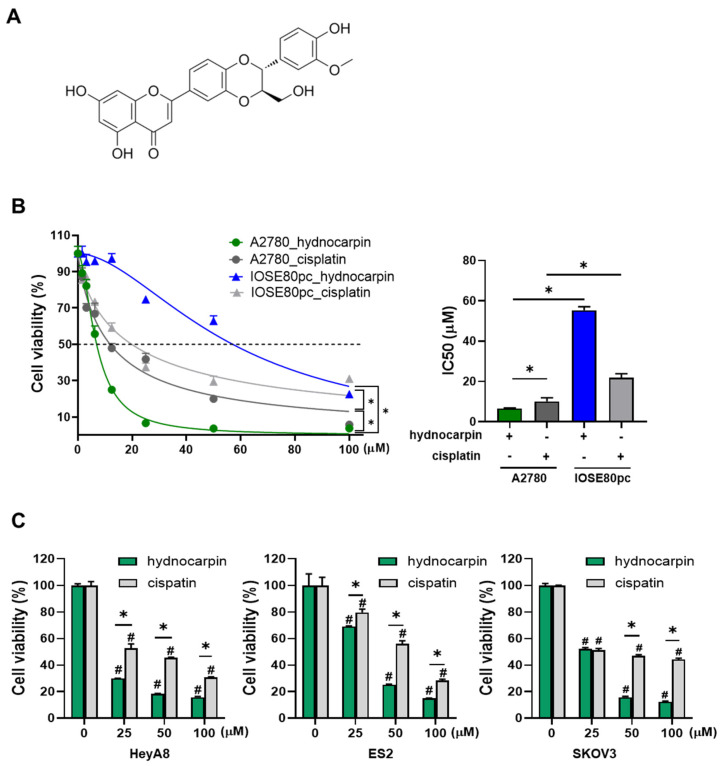
Hydnocarpin isolated from *Pueraria Flos* (PF) inhibits the growth of human ovarian cancer cells. (**A**) Chemical structure of hydnocarpin. (**B**) A2780 ovarian cancer cells and IOSE80pc immortalized ovarian surface epithelial cells were treated with the indicated concentration of hydnocarpin or cisplatin for 48 h. Cell viability was assessed using the MTT assay (**left**) and the half-maximal inhibitory concentration (IC50) values were calculated (**right**). (**C**) HeyA8, ES2, and SKOV3 ovarian cancer cell lines were treated with varying concentrations of hydnocarpin or cisplatin for 48 h, and cell viability was measured by MTT assay. Data are representative of three independent experiments. # *p* < 0.05 compared with the untreated control group. * *p* < 0.05 compared with the indicated group.

In our previous study, hydnocarpin (Figure 1A), among 22 compounds isolated from the methanolic extract of PF, exhibited the most pronounced inhibitory effect on the growth of A2780 human ovarian cancer cells [14]. As shown in Figure 1B, hydnocarpin significantly suppressed the growth of A2780 cells and exhibited stronger cytotoxicity than cisplatin, a commonly used chemotherapeutic agent for ovarian cancer. Cisplatin exerted comparable cytotoxic effects against the A2780 and immortalized ovarian surface epithelial cells; however, hydnocarpin showed markedly lower toxicity against the ovarian surface epithelial cells, indicating its high tumor selectivity. Consistently, hydnocarpin exerted growth-inhibitory effects superior to those of cisplatin on ovarian cancer cell lines, including the SKOV3, ES2, and HeyA8 lines (Figure 1C).

**Figure 2:**
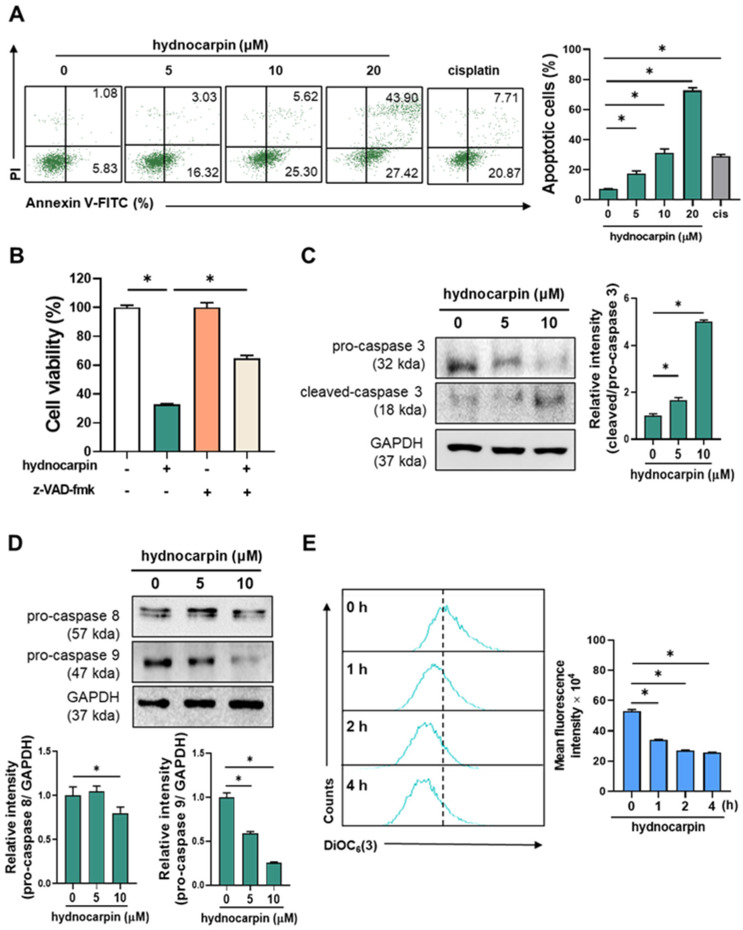
Hydnocarpin induces the caspase-dependent apoptosis of human ovarian cancer cells. (**A**) A2780 cells were treated with various concentrations of hydnocarpin (5, 10, 20 μM) or 20 μM of cisplatin for 48 h, followed by Annexin V-FITC/PI double staining and flow cytometry analysis to assess apoptotic cell populations. (**B**) A2780 cells were pretreated with the pan-caspase inhibitor z-VAD-fmk (100 μM) for 2 h prior to 48 h exposure to 10 μM of hydnocarpin. Cell viability was evaluated using the MTT assay. (**C**,**D**) Western blot analysis of pro- and cleaved forms of caspase-3 (**C**) and pro-caspase-8/9 (**D**) in A2780 cells after 48 h hydnocarpin treatment and densitometric analysis of the Western blot bands. (**E**) Cells were treated with 10 μM of hydnocarpin for the indicated times, stained with DiOC_6_(3). Data are representative of three independent experiments. * *p* < 0.05 compared with the indicated group.

Next, annexin V-fluorescein isothiocyanate/propidium iodide double staining was performed to determine whether hydnocarpin-induced growth inhibition is associated with apoptosis. As shown in Figure 2A, hydnocarpin (5, 10, and 20 μM) significantly increased the number of apoptotic A2780 cells in a dose-dependent manner. Notably, cisplatin (positive control) induced a lower level of apoptosis than hydnocarpin at the same concentration (20 μM). These results suggest that hydnocarpin exerts more potent anticancer effects than cisplatin by promoting cell apoptosis. To further elucidate the underlying apoptotic mechanisms, we examined the roles of caspases, the key apoptosis regulators [25], in hydnocarpin-induced cell death. As shown in Figure 2B, pretreatment with the pan-caspase inhibitor, z-VAD-fmk, significantly reversed the hydnocarpin-induced cytotoxicity, including caspase-dependent cell apoptosis. 

Western blotting analysis demonstrated that hydnocarpin markedly decreased the levels of pro-caspase-3 and increased cleaved caspase-3 expression (Figure 2C). A pronounced reduction in pro-caspase-9 was also observed, whereas pro-caspase-8 levels remained largely unchanged (Figure 2D). These findings suggest that the intrinsic, mitochondrial-mediated apoptotic pathway—governed by caspase-9—is critically involved in hydnocarpin-induced apoptosis [26]. To further confirm mitochondrial involvement, we assessed mitochondrial membrane potential. As shown in Figure 2E, treatment with hydnocarpin led to a significant loss of mitochondrial membrane potential, indicating mitochondrial depolarization. Taken together, these results support the conclusion that hydnocarpin induces apoptosis in ovarian cancer cells primarily through the caspase-9-dependent intrinsic mitochondrial pathway, rather than the extrinsic death receptor pathway.

### 3.2. Hydnocarpin-Induced Ovarian Cancer Cell Apoptosis Involves Reactive Oxygen Species (ROS) Production and NADPH Oxidase (NOX) Activation

**Figure 3:**
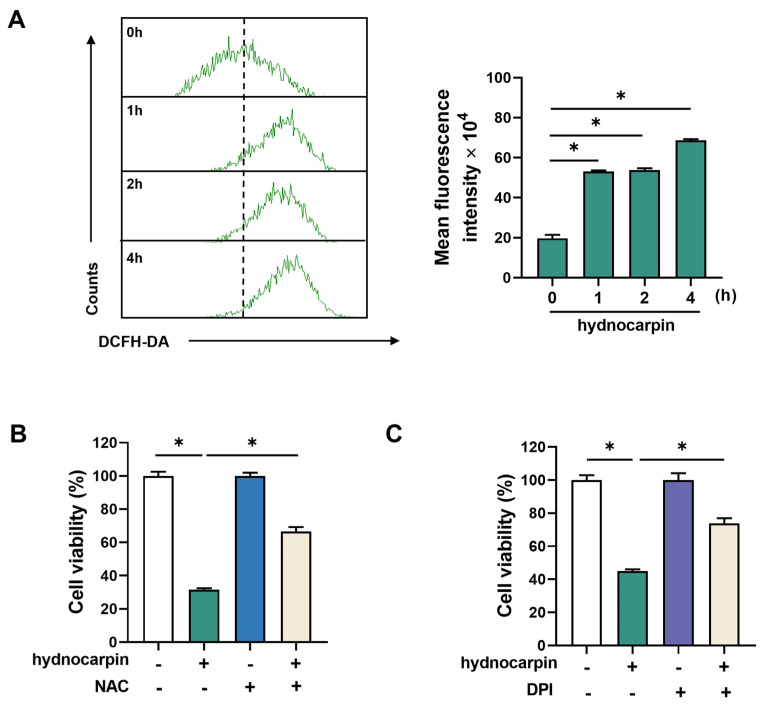
Roles of reactive oxygen species (ROS) and NADPH oxidase in the hydnocarpin-induced apoptosis of ovarian cancer cells. (**A**) A2780 cells were exposed to 10 μM of hydnocarpin for 0, 2, or 4 h, followed by staining with 1 μM of DCFH-DA for 30 min. The intensity of fluorescence was analyzed by flow cytometry. (**B**) A2780 cells were pretreated with NAC (7.5 mM) for 30 min prior to treatment with 10 μM of hydnocarpin for 48 h. Cell viability was assessed by MTT assay. (**C**) Cells were pretreated with DPI (0.1 μM), a NOX inhibitor, for 30 min, before exposure to 10 μM of hydnocarpin for 48 h. Cell viability was assessed by MTT assay. Data are representative of three independent experiments. * *p* < 0.05 compared with the indicated group.

As mitochondria are recognized as the primary source of intracellular ROS production [27], and ROS has been implicated in mediating apoptosis in ovarian cancer cells [28], we investigated whether ROS generation is involved in hydnocarpin-induced cytotoxicity. As shown in Figure 3A, hydnocarpin time-dependently increased the intracellular ROS levels in A2780 cells, as determined by the 2′,7′-dichlorodihydrofluorescein diacetate staining and flow cytometry assays. To assess whether ROS elevation plays a critical role in hydnocarpin-induced cell death, A2780 cells were pretreated with N-acetylcysteine, a well-known antioxidant. N-acetylcysteine pretreatment significantly reversed the cytotoxic effects of hydnocarpin, suggesting ROS generation as a key mediator of the hydnocarpin apoptotic activity (Figure 3B). This ROS-dependent cytotoxicity was further validated in additional ovarian cancer cell lines, SKOV3 and ES2, where NAC similarly rescued cell viability (Supplementary Figure S1).

NOX is a major source of intracellular ROS in cancer cells [29]. To determine whether NOX contributes to hydnocarpin-induced ROS production, A2780 cells were pretreated with the NOX inhibitor, diphenyleneiodonium chloride. As illustrated in Figure 3C, pretreatment with diphenyleneiodonium chloride significantly inhibited hydnocarpin-induced cell death, suggesting the critical role of NOX activation in hydnocarpin-induced ROS-mediated apoptosis. Collectively, these findings suggest that hydnocarpin induces ovarian cancer cell apoptosis via a NOX-dependent ROS production pathway.

### 3.3. Hydnocarpin Reprograms the Ovarian Cancer-Stimulated MQs (OC-MQs) by Suppressing the Tumor-Promoting Genes and Enhancing the Phagocytic Activity

**Figure 4:**
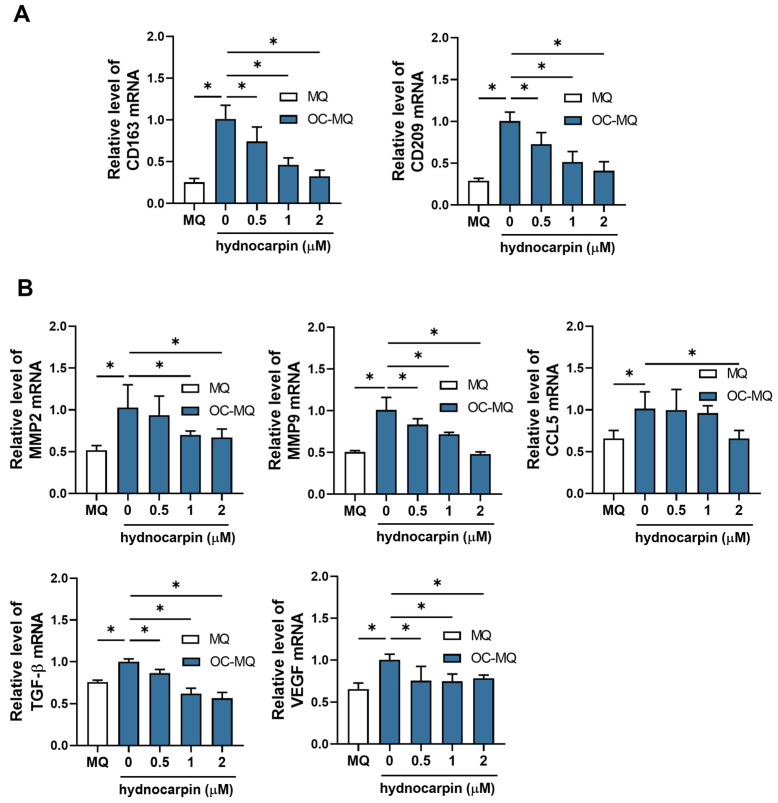
Effects of hydnocarpin on tumor-promoting factors and phagocytic activity of tumor-associated macrophages. THP-1-derived macrophages (MQs) were exposed to conditioned medium (CM) from A2780 cells for 24 h and subsequently treated with hydnocarpin for an additional 48 h. (**A**) Expressions of M2 macrophage markers (CD163 and CD209) were measured via RT-PCR. (**B**) mRNA levels of pro-tumoral genes (MMP-2/-9, CCL5, TGF-β, and VEGF) were quantified via RT-PCR. Dark-blue bars indicate OC-MQs and white bars indicate MQs. Data are representative of three independent experiments. * *p* < 0.05 compared with the indicated group.

Tumor-associated MQs (TAMs), the TME components around tumors, interact with cancer cells [30]. These MQs are polarized toward the pro-tumor M2 phenotype by cancer cell-derived factors, resulting in the loss of their phagocytic activity and the promotion of tumor progression [31]. To investigate the effects of hydnocarpin on TAMs, we established OC-MQs by treating MQs with the conditioned medium (CM) of A2780 cells. As shown in Figure 4A, hydnocarpin significantly reduced the expression levels of the M2 MQ markers, CD163 and CD209, suggesting that hydnocarpin reprograms the OC-MQs. Furthermore, hydnocarpin markedly decreased the expression levels of cancer-promoting factors, including matrix metalloproteinase (MMP)-2/9, C–C motif chemokine ligand 5, transforming growth factor-β, and vascular endothelial growth factor, which were upregulated in OC-MQs (Figure 4B). These findings suggest that hydnocarpin counteracts the tumor-promoting phenotype of OC-MQs.

**Figure 5:**
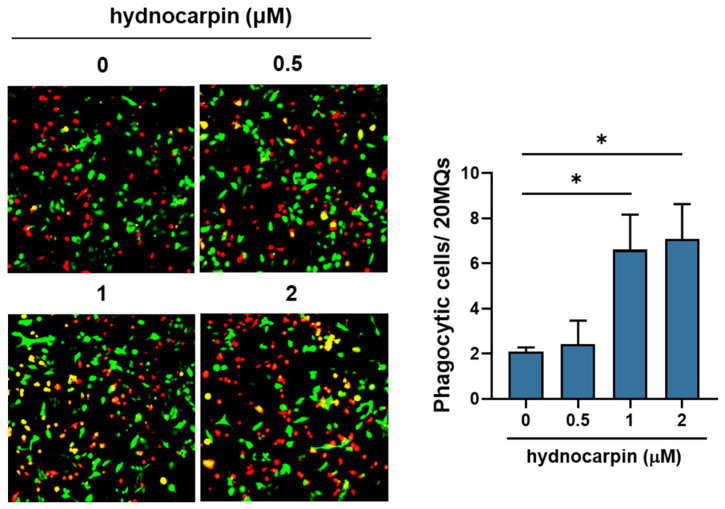
Effect of hydnocarpin on the phagocytic activity of ovarian cancer-stimulated macrophages. CellTracker-Red-labeled MQs were co-cultured with CellTracker-Green-labeled A2780 cells in the presence of hydnocarpin for 48 h. Phagocytosis was assessed by counting GFP-positive phagocytic MQs per 20 MQs. Data are representative of three independent experiments. * *p* < 0.05 compared with the indicated group.

In MQ co-cultures with A2780 cells, hydnocarpin significantly increased the phagocytic activity of MQs in A2780 cells in a dose-dependent manner (Figure 5). Collectively, our findings suggest that hydnocarpin reprograms OC-MQs by downregulating the tumor-promoting gene levels and enhancing their tumor-clearing activity.

### 3.4. Hydnocarpin Modulates the Immune Evasion-Related Genes in the Tumor-Associated Immune Cells

**Figure 6:**
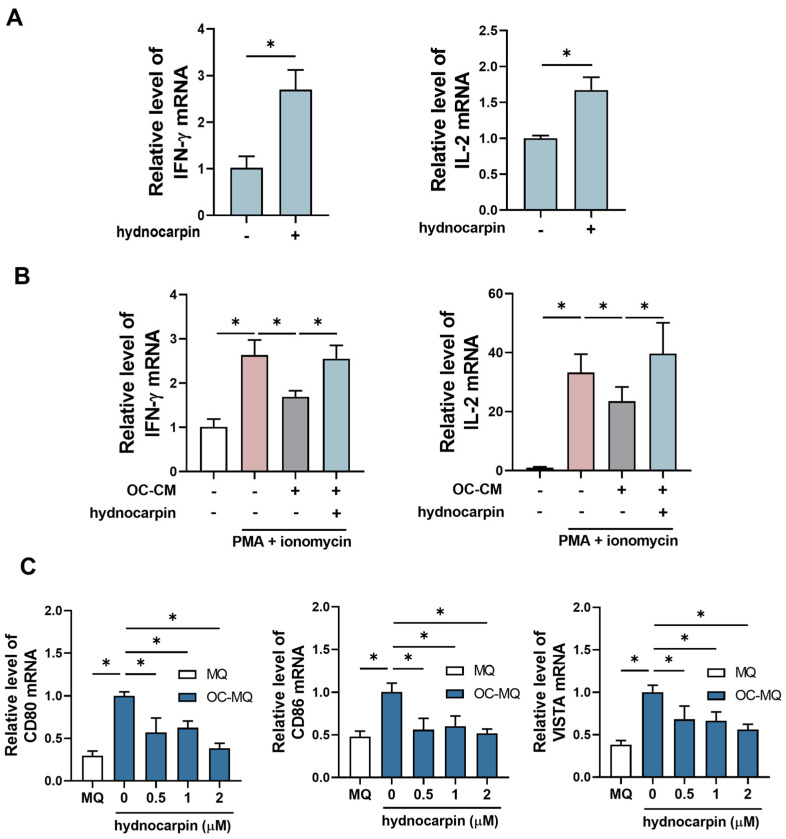
Effect of PF-isolated hydnocarpin on T-cell activation in the tumor microenvironment. RT-PCR was performed to assess the mRNA levels of IFN-γ and IL-2. (**A**) CCRF-HSB2 cells were treated with 2 μM of hydnocarpin for 48 h. (**B**) CCRF-HSB2 cells activated by PMA and ionomycin were stimulated with the CM of A2780 (OC-TCs) and 2 μM of hydnocarpin for 48 h. (**C**) RT-PCR was conducted to evaluate the mRNA levels of immune suppression-related genes (CD80, CD86, and VISTA) in OC-MQs. Dark-blue bars indicate OC-MQs and white bars indicate MQs. Data are representative of three independent experiments. * *p* < 0.05 compared with the indicated group.

The low activation of T cells is a critical immune evasion mechanism in cancer [32]. To evaluate whether hydnocarpin influences T-cell activation, we determined the expression levels of the key T-cell activation-associated cytokines, interferon (IFN)-γ and interleukin (IL)-2, in T cells (CCRF-HSB2). As shown in Figure 6A, hydnocarpin significantly upregulated the IFN-γ and IL-2 mRNA levels. Under T-cell activation conditions (ovarian cancer-stimulated T cells [OC-TCs] induced by PMA and ionomycin), the CM of A2780 cells (OC-CM) suppressed IFN-γ and IL-2 expression in CCRF-HSB2 cells. However, hydnocarpin restored the expression of these cytokines in OC-TCs (Figure 6B), showing potential to counteract tumor-induced immune suppression. TAMs regulate immune evasion by modulating the T-cell co-stimulatory and checkpoint molecules, such as CD80, CD86, and VISTA [33,34], as shown in Figure 6C. OC-MQs exhibited elevated CD80, CD86, and VISTA expression levels; however, these levels were significantly reduced by hydnocarpin in a dose-dependent manner. These results suggest that hydnocarpin alleviates immune suppression in TME by modulating the TAM-mediated T-cell regulatory molecule expression. Overall, hydnocarpin enhanced T-cell activation and simultaneously reduced the immune evasion-associated gene expression in TAMs, showing dual roles in immunomodulation and tumor suppression.

## 4. Discussion

Several PF-derived isoflavonoids exhibit anticancer activities [8]. For example, Chen et al. revealed six PF-derived isoflavonoids (6′-O-xylosylglycitin, tectorigenin, 6′-O-xylosyltectoridin, glycitein, tectoridin, and glycitin), among which tectorigenin induced both the differentiation and apoptosis of leukemia cells [35]. Hydnocarpin, a flavone-type flavonolignan, exerts anti-tumor effects against various malignancies [14,16,20,21,22]. Specifically, it inhibits the proliferation of colon cancer cells [20], suppresses the malignant progression of triple-negative breast cancer cells [21], and reduces the growth of various tumor cell lines, including nasopharyngeal carcinoma, colon adenocarcinoma, osteosarcoma, uterine carcinoma, and glioma cell lines [16]. Moreover, hydnocarpin enhances the drug sensitivity of multiple drug-resistant cancer cell lines [22]. However, to date, most studies have primarily focused on its direct cytotoxic effects, with limited exploration of its underlying molecular mechanisms and immunomodulatory roles.

To the best of our knowledge, this study is the first to demonstrate the involvement of ROS in the anticancer effects of hydnocarpin on ovarian cancer cells and its modulatory effects on tumor-associated immune cells. ROS play multifaceted roles in cancer biology, including the regulation of cell cycle progression, proliferation, differentiation, migration, and apoptosis [28]. In this study, hydnocarpin induced ovarian cancer cell apoptosis by activating the caspase-9-dependent intrinsic pathway mediated via ROS production. These findings were confirmed by network pharmacological predictions (Supplementary Figure S2). The top 100 proteins targetable by hydnocarpin were selected using the SwissTargetPrediction database. Simultaneously, 1310 targets associated with ovarian cancer were identified through the GeneCards database search. A Venn diagram revealed 30 overlapping targets between hydnocarpin and ovarian cancer, including key genes such as *MMP2* and *MMP9*. The Gene Ontology enrichment analysis of biological processes revealed significant enrichment in the oxidative stress pathways, including the “cellular response to oxidative stress” and “oxidative stress” pathways (Supplementary Table S2). Consistently, a previous study also showed hydnocarpin-induced ROS accumulation and apoptosis in leukemia cells [22], highlighting the pro-oxidant roles of hydnocarpin in cancer cells. Additionally, hydnocarpin derivatives induce the ROS-mediated apoptosis of lung cancer and melanoma cells [36]; this suggests the core structure involvement in the pro-oxidant and pro-apoptotic effects of hydnocarpin. Collectively, our findings suggest that hydnocarpin induces the ROS-mediated apoptosis of human ovarian cancer cells, warranting the further investigation of its potential as a ROS-targeting anticancer agent.

Furthermore, NADPH oxidases (NOXs) are recognized as primary sources of intracellular ROS production [29], and our findings support that hydnocarpin induces apoptosis in ovarian cancer cells by activating the caspase-9-dependent intrinsic apoptotic pathway via NOX-mediated ROS generation. While our study confirmed the involvement of NOX in ROS production, the specific isoforms responsible were not experimentally identified. Nonetheless, previous studies have demonstrated that NOX2 and NOX4 are frequently upregulated in ovarian cancer and play pivotal roles in tumor progression, chemoresistance, and redox homeostasis [37,38,39,40]. For example, NOX4 overexpression has been correlated with enhanced ROS production, epithelial-to-mesenchymal transition, and poor clinical outcomes in ovarian cancer patients [37]. An analysis of TCGA transcriptomic data revealed high expression levels of NOX2 and NOX4 in ovarian cancer tissues (Supplementary Figure S3A), with significantly greater expression in tumor samples compared to normal ovarian tissues (Supplementary Figure S3B). Furthermore, our molecular docking study suggests that hydnocarpin may interact directly with NOX2 and NOX4, showing weak-to-moderate binding affinities (Supplementary Table S3). These observations raise the possibility that hydnocarpin-induced ROS production and subsequent apoptotic signaling may be partially mediated through the modulation of NOX2 or NOX4 activity. Taken together, although further experimental validation (e.g., isoform-specific knockdown) is required, our findings and the existing literature suggest that NOX2 and NOX4 are likely contributors to the ROS-mediated apoptotic effects of hydnocarpin in ovarian cancer cells.

The targeting of both tumor cells and TME, including TAMs and T cells, is necessary for effective ovarian cancer treatment [41,42]. TAMs interact with other TME components to support tumor growth and metastasis [30,43]. An increase in C–C motif chemokine ligand 5, MMP-2/9, transforming growth factor-β, and vascular endothelial growth factor levels by TAMs promotes cancer cell invasion, epithelial–mesenchymal transition, and angiogenesis, respectively [44,45,46]. In this study, hydnocarpin suppressed the expression of these pro-tumor genes in OC-MQs, modulating the TME toward an anti-tumor state. Furthermore, because TAMs represent a key phagocytic population in tumors, restoring their phagocytic activity offers a therapeutic advantage, as tumor cells often evade MQ-mediated clearance [47]. In this study, hydnocarpin significantly enhanced the phagocytic activity of OC-MQs in a dose-dependent manner. These findings suggest that hydnocarpin suppresses tumor-supporting gene expression in TAMs and reprograms them toward an anti-tumor phenotype.

T cells in TME often lose their cytotoxic activity due to immunosuppressive signaling [42]. Therefore, T-cell activity restoration is a promising strategy against immune evasion. TAMs contribute to T-cell dysfunction by expressing immunosuppressive molecules, such as CD80, CD86, and VISTA [33,34], which inhibit T-cell activation and impair the anti-tumor immunity. In this study, hydnocarpin downregulated the CD80, CD86, and VISTA mRNA levels in OC-MQs, thereby alleviating immune suppression and restoring the T-cell functions in the TME. These data suggest that hydnocarpin contributes to anti-tumor immunity reactivation by modulating the TAM–T-cell crosstalk. Interestingly, hydnocarpin did not affect the expression of programmed death-ligand 1, a key immune checkpoint molecule commonly upregulated in TAMs and associated with immune evasion [48], as shown in our study (Supplementary Figure S4). This differential modulation suggests that hydnocarpin may selectively target specific immunoregulatory pathways. Notably, CD80, CD86, and VISTA are transcriptionally regulated by signaling cascades involving NF-κB and STAT3, both of which are known to influence macrophage function and immune activation [49,50,51]. By contrast, PD-L1 expression is predominantly induced by the IFN-γ signaling pathway [52,53]. Thus, the selective downregulation of CD80, CD86, and VISTA, but not PD-1, may reflect the compound’s pathway-specific effects, likely mediated through its suppression of NF-κB or STAT3 activity rather than IFN-γ-driven transcriptional mechanisms. However, further mechanistic studies, including analyses of upstream transcriptional and epigenetic regulators, will be needed to fully elucidate this selective effect.

Flavonolignans represent a unique class of phenolic compounds, combining flavonoid and lignan structural motifs. Notably, other flavonolignans have also demonstrated immunomodulatory effects. For instance, salcolin B and salcolin C, isolated from *Oryza sativa*, significantly inhibited nitric oxide production in LPS-stimulated RAW 264.7 macrophages, without exhibiting cytotoxicity [54]. Similarly, *Hippophae*-derived flavonolignans were recently shown to exert immunosuppressive and neuroprotective effects in vitro, modulating microglial responses [55,56]. These findings underscore the inherent immunomodulatory capabilities of flavonolignans. In contrast, our study provides the first evidence that hydnocarpin may not only attenuate pro-tumoral M2 macrophage phenotypes but also enhance macrophage-mediated phagocytosis and restore T-cell activation within the tumor microenvironment. This dual modulation of both innate and adaptive immunity distinguishes hydnocarpin from previously characterized flavonolignans, offering a novel therapeutic avenue combining ROS-mediated tumor cell killing with immune system reprogramming.

In addition to its anticancer efficacy, the potential systemic and immunotoxicity of hydnocarpin warrants careful consideration. ADMET prediction tools have suggested the toxicological profile of hydnocarpin, predicting low risks for skin, renal, and mutagenic toxicity, while indicating possible concerns related to hepatotoxicity and reproductive toxicity (https://plantaedb.com/) [57]. However, experimental evidence supports its relatively selective cytotoxicity toward cancer cells. For example, hydnocarpin showed minimal cytotoxicity in normal human ovarian epithelial cells (IOSE80PC) as well as in immune-related cell types such as CCRF-HSB2 T lymphocytes and THP-1-derived macrophages in our study (Supplementary Figure S5). Similarly, prior studies have demonstrated that hydnocarpin does not compromise the viability of other T-cell acute lymphoblastic leukemia cell lines (697, Jurkat, and Molt-4) or normal human lymphocytes (CAM-191) at comparable concentrations [18,58]. Furthermore, RAW264.7 macrophages [17] and AML12 hepatocytes [22] displayed much higher IC_50_ values than cancer cells, supporting a favorable therapeutic index. An in vivo toxicity study of *Hydnocarpus wightiana* seed extract, which contains hydnocarpin, reported no significant adverse effects following the oral administration of doses up to 5000 mg/kg in rodents [59], further reinforcing its safety profile. Although these findings suggest that hydnocarpin exerts potent anticancer effects with limited off-target toxicity—particularly toward immune cells—further in vivo validation, including systemic toxicity and detailed immunotoxicological assessments, is essential to support its clinical translation.

## 5. Conclusions

In conclusion, this study revealed the dual anticancer effects of hydnocarpin on ovarian cancer cells, including its direct effects via ROS-mediated cell apoptosis and indirect effects via the modulation of tumor-associated immune cells, including TAMs and T cells. Our findings highlight hydnocarpin as a promising therapeutic agent for ovarian cancer. However, further studies are necessary to determine the specific action mechanisms and in vivo efficacy of hydnocarpin.

## Data Availability

This study utilized publicly available datasets, as cited in the manuscript. All other data generated or analyzed during the current study are included in this published article and its Supplementary Information Files.

## Supplementary Materials

The following supporting information can be downloaded at: https://www.mdpi.com/article/10.3390/antiox14070846/s1, Figure S1: Effect of NAC on the hydnocarpin-induced apoptosis in human ovarian cancer SKOV3 and ES2 cells; Figure S2: Network pharmacology and Gene Ontology (GO) enrichment analysis for the identification of hydnocarpin and ovarian cancer-related targets and its enriched pathways; Figure S3: Expression of NOX family genes in normal and cancerous ovarian tissues; Figure S4: Effect of hydnocarpin on PD-L1 expression in tumor-associated macrophages; Figure S5: Effect of hydnocarpin on cell viability in CCRF-HSB2 cells and macrophages; Table S1: Primer sequences used for RT-PCR; Table S2: Top 20 Biological Processes Identified by GO Analysis Based on *p*-values; Table S3: Binding affinity of hydnocarpin on NOX2 and NOX4 [60,61,62,63].

## Abbreviations

TME	Tumor microenvironment
PF	*Pueraria* *F* *los*
TAMs	Tumor-associated macrophages
ROS	Reactive oxygen species
CM	Conditioned media
MQs	Macrophages
OC-MQs	Ovarian cancer-stimulated macrophages
OC-TCs	Ovarian cancer-stimulated T cells
