# Supplementary material for: Hydnocarpin, a Natural Flavonolignan, Induces the ROS-Mediated Apoptosis of Ovarian Cancer Cells and Reprograms Tumor-Associated Immune Cells
Source: Antioxidants (Basel). 2025 Jul 10;14(7):846. doi: 10.3390/antiox14070846 (PMC12292001; doi:10.3390/antiox14070846)
Supplement: Supplementary file 1 [file antioxidants-14-00846-s001.zip › antioxidants-3691123-supplementary.pdf]

Supplementary Information

“Hydnocarpin, a natural flavonolignan, induces the ROS-mediated apoptosis of ovarian cancer cells and reprograms tumor-associated immune cells”

Kim JY et al.

Supplementary Methods

Network pharmacological analysis

Predicted targets of hydnocarpin were collected from SwissTargetPrediction (www.swisstargetprediction.ch). Ovarian cancer-associated genes were obtained from the GeneCards (https://www.genecards.org) at the threshold of relevance score as 17.5. The protein–protein interaction (PPI) network was constructed using the STRING application and Cytoscape (version 3.10.1), with the minimum required interaction score set at a high confidence level (0.700). Based on the STRING PPI network, we took topological analysis by Analyze Network tool of Cytoscape to find hub target proteins. We used the R software (version 4.3.1) to perform gene ontology (GO) enrichment and visualized it by a bar plot and a dot plot.

Expression of NOX subtype in human ovarian tissues

Transcriptomic data from ovarian cancer tissues (TCGA-OV, n=427) and normal ovarian tissues (GTEx, n=88) were retrieved and compared using the UCSC Xena Browser (https://xenabrowser.net). The merged dataset, "TCGA TARGET GTEx (TOIL) gene expression," which includes uniformly processed RNA-seq data from both TCGA and GTEx cohorts, was used to evaluate the relative mRNA expression levels of NOX family genes. Samples were filtered by tissue type ("Ovary") and sample type ("Primary Tumor" for TCGA-OV and "Solid Tissue Normal" for GTEx). The dataset used was based on RNA-seq data quantified and normalized using the RSEM method. Data visualization and figure generation were performed using GraphPad Prism 8 software (San Diego, CA, USA). and statistical significance between cancer and normal tissues was assessed using an unpaired two-tailed Student's t-test.

Molecular docking study

The three-dimensional structures of NOX2 (UniProt ID: P04839) and NOX4 (UniProt ID: Q9NPH5) were obtained from the AlphaFold Protein Structure Database (https://alphafold.ebi.ac.uk/). Molecular docking was performed using SwissDock

(http://www.swissdock.ch/)[1,2] based on Autodock Vina [3,4] was used for performing molecular docking simulations. The top 10 ranked binding poses based on estimated binding affinity were used.

Cell counting kit-8 (CCK-8) assay

CCRF-HSB2 cells were transfected with siRNA or ASO using the aforementioned transfection reagent. After 24 hours, CCK-8 solution (GLPBIO, Montclair, CA, USA) was introduced to each well, and the plates underwent an additional 1-2 hours of incubation at 37°C. Then, the cell viability was determined by measuring the optical density absorbance at the wavelength of 450 nm.

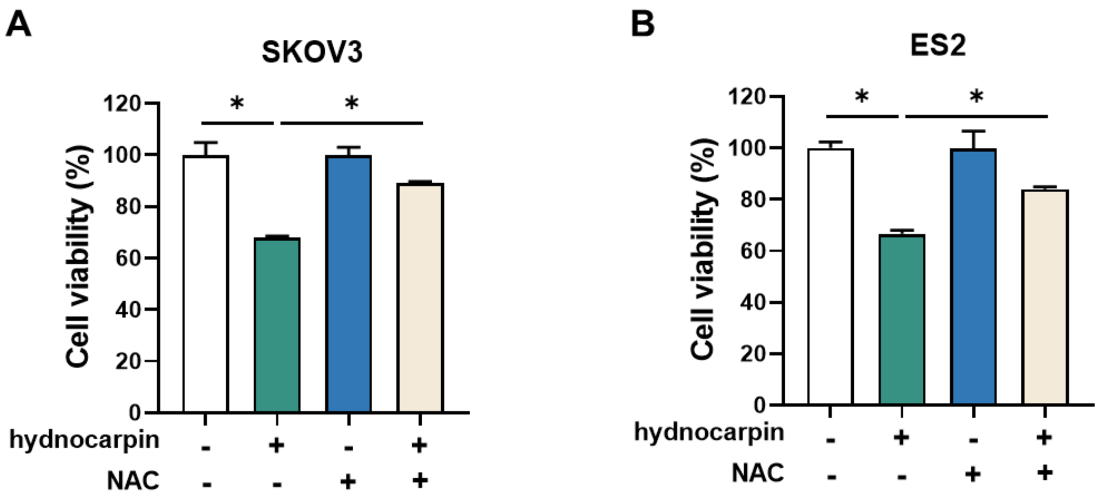

Supplementary Figure S1. Effect of NAC on the hydnocarpin-induced apoptosis in hu man ovarian cancer SKOV3 and ES2 cells

SKOV3 (A) and ES2 (B) cells were pretreated with NAC (7.5 mM) for 30 minutes prior to treatment with 25 μM hydnocarpin for 48 hours. Cell viability was assessed by MTT assay. Data are representative of three independent experiments. * $p < 0.05$ compared with the indicated group.

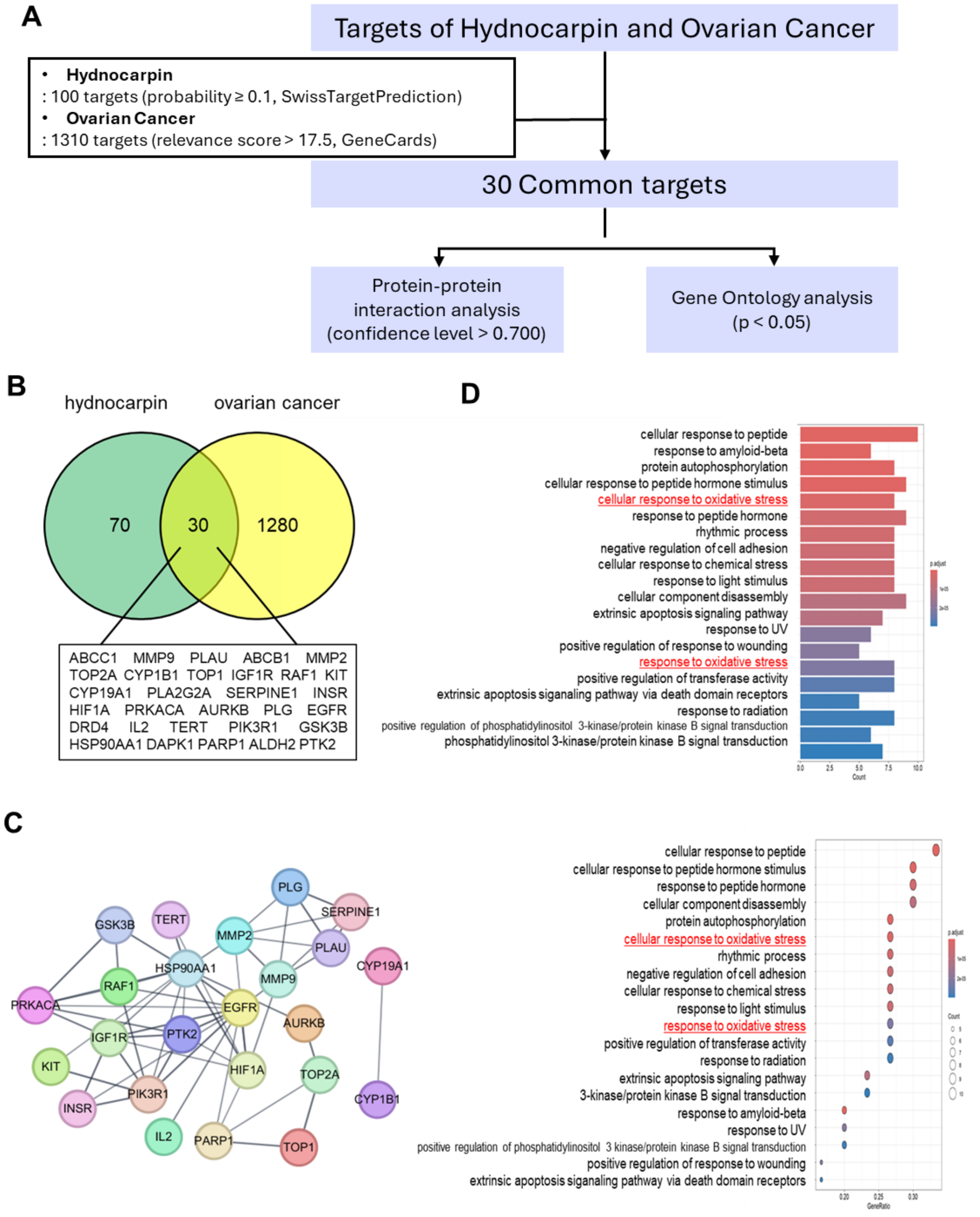

Supplementary Figure S2. Network pharmacology and Gene Ontology (GO) enrichment analysis of hydnocarpin target in ovarian cancer.

(A) Scheme image of the analysis workflow (B) Venn diagram showing the intersection between predicted hydnocarpin targets (green, n=100) and ovarian cancer genes obtained from GeneCards (yellow, n=1,310; relevance score > 17.5) (C) Protein-protein interaction (PPI)

network of the 30 common targets was constructed using STRING (confidence score > 0.700), (D) GO analysis plots for biological processes, with the top 20 components ($p < 0.05$) on the Y-axis, and the number of genes (bar plot) and gene ratios (dot plot) on the X-axis related to these processes.

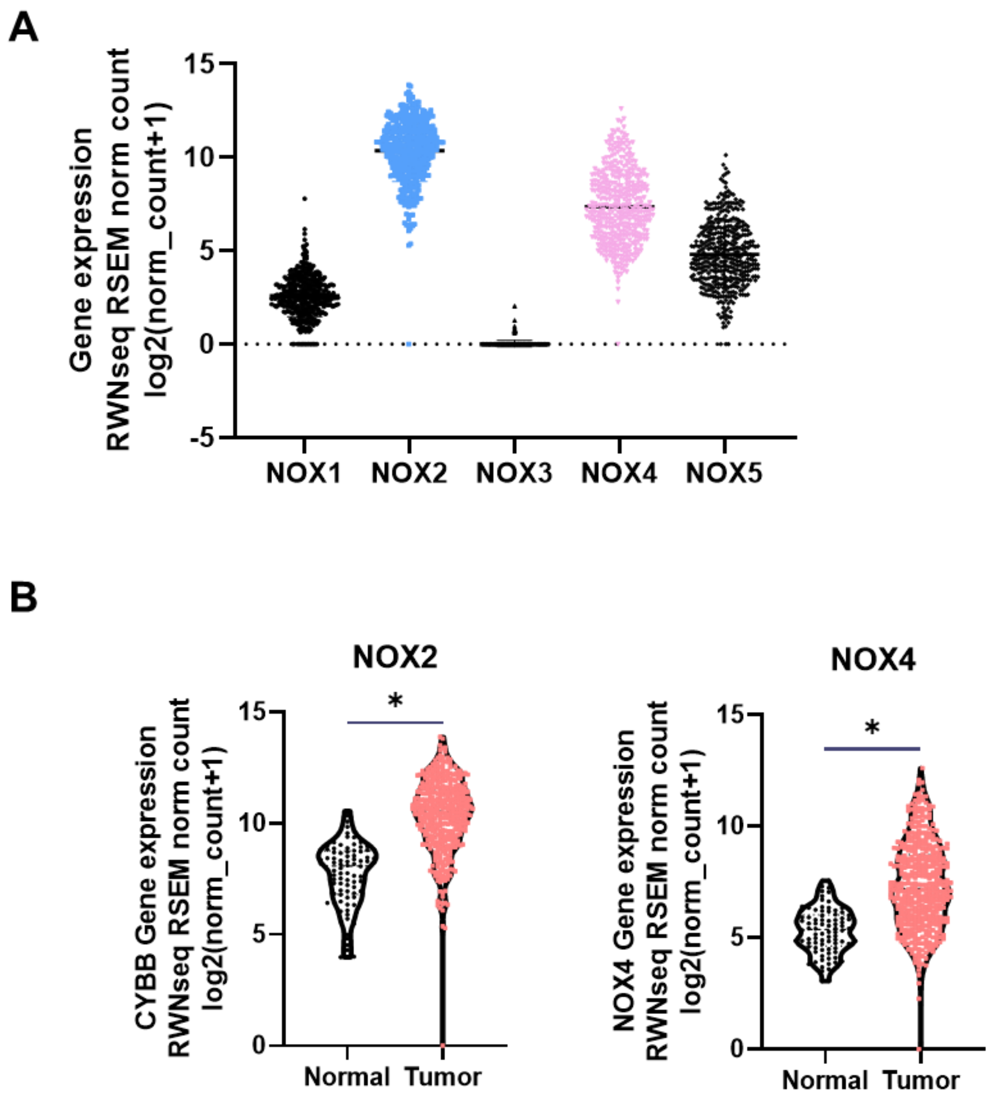

Supplementary Figure S3. Expression of NOX family genes in normal and cancerous ovarian tissues

(A) Gene expression levels of NOX family members (NOX1–NOX5) in ovarian cancer samples from the TCGA-OV dataset. RNA-seq expression data were obtained from the TCGA-OV cohort and normalized using RSEM. (B) The mRNA expression levels of NOX2 (CYBB) and NOX4 were compared between normal ovarian tissues and ovarian cancer tissues. Gene expression data were obtained from the UCSC Xena Browser using the TCGA TARGET GTEx (TOIL) gene expression dataset. Normal ovarian tissue samples (GTEx, =88) and primary ovarian cancer tissue samples (TCGA-OV, n=427) were analyzed. * $p < 0.05$ compared with the indicated group;

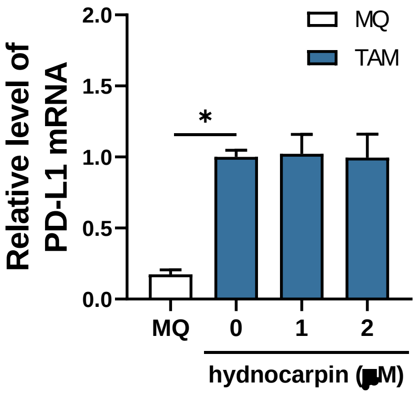

Supplementary Figure S4. Effect of hydnocarpin on PD-L1 expression in tumor-associated macrophages

THP-1 cells were stimulated with conditioned medium (CM) from A2780 cells for 24 hours and were treated with the indicated concentration of hydnocarpin (1 and 2 μM) for 48 h. Real-time RT-PCR was conducted to measure the mRNA levels of PD-L1 in THP-1 with or without CM of A2780. The blue bars in the graph represent THP-1 stimulated by the CM of A2780. The data are representative of three independent experiments. * $p < 0.05$ compared with indicated group

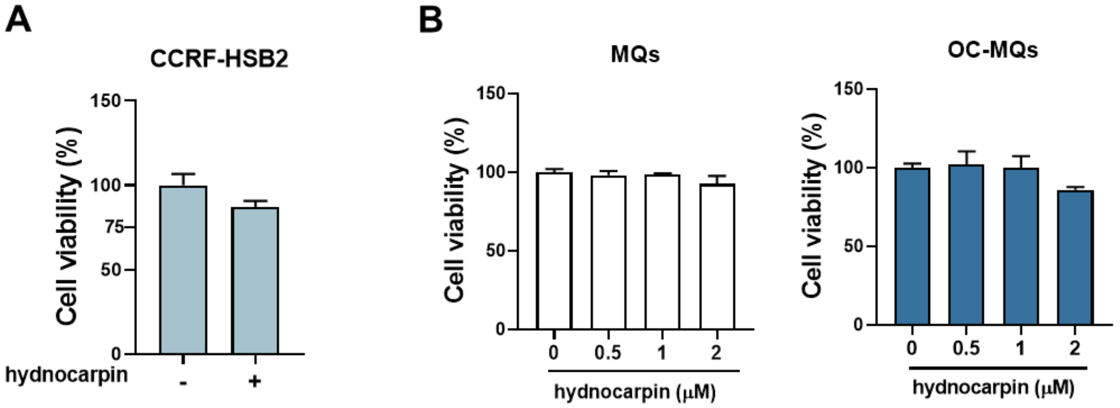

Supplementary Figure S5. Effect of hydnocarpin on cell viability in CCRF-HSB2 and macrophages.

(A) CCRF-HSB2 cells were treated with 2 μM hydnocarpin for 48 hours. CCK-8 assay was conducted to evaluate the cell viability. (B) THP-1-derived macrophages (MQs) were exposed to conditioned medium from A2780 cells for 24 hours (OC-MQs) and subsequently treated with hydnocarpin for an additional 48 hours. Cell viability was assessed by MTT assay. Data are representative of three independent experiments.

Supplementary Table S1. Primer sequences used for RT-PCR

	Forward (5’→3’)	Reverse (5’→3’)
CD163	AGC AGG GAT GTT GGA GTA GT	TAA GCT GCT GGC AAA GAA CA
CD209	CCA GGA TGG TCT CGA TCT CT	CAG CGA GGA AGA AAC CTA CC
MMP-2	TGA TCT TGA CCA GAA TAC CAT CGA	GGC TTG CGA GGG AAG AAG TT
MMP-9	GGA TAC AGT TTG TTC CTC GT	GTA CAT AGG GTA CAT GAG CG
TGF-β	CGT GGA GCT GTA CCA GAA ATA C	CAC AAC TCC GGT GAC ATC AA
VEGF	ATG GCA GAA GGA GGA GGG CA	ATC GCA TCA GGG GCA CAC AG
CD80	GAC GAG GGC ACA TAC GAG TG	AAC GTC ACT TCA GCC AGG TG
CD86	ATT CGG ACA GTT GGA CCC TG	CCA AGG AAT GTG GTC TGG GG
RANTES	GGG TTC GGG AGT ACA TCA AC	CTG TGT GGT AGA ATC TGG GC
VISTA	GAT AGC GGC CTC TAC TGC TG	TGG ATG GTG CAT CTT TGC CT
CD80	GAC GAG GGC ACA TAC GAG TG	AAC GTC ACT TCA GCC AGG TG
CD86	ATT CGG ACA GTT GGA CCC TG	CCA AGG AAT GTG GTC TGG GG
VISTA	AGA CAG GCA AAG ATG CAC CA	TGC AGC CGT GAT GTT TTC AC
β-actin	CAA ACA TGA TCT GGG TCA TC	GCT CGT CGT CGA CAA CGG CT

Supplementary Table S2. Top 20 Biological Processes Identified by GO Analysis Based on P-values.

no.	GO ID	Description	Enriched genes
1	GO:1901653	cellular response to peptide	ABCC1/CYP1B1/RAF1/GSK3B/PARP1/PRKACA/IGF1R/INSR/PIK3R1/PTK2
2	GO:1904646	response to amyloid-beta	ABCC1/GSK3B/PARP1/MMP9/MMP2/IGF1R
3	GO:0046777	protein autophosphorylation	GSK3B/KIT/EGFR/IGF1R/INSR/AURKB/DAPK1/PTK2
4	GO:0071375	cellular response to peptide hormone stimulus	CYP1B1/RAF1/GSK3B/PARP1/PRKACA/IGF1R/INSR/PIK3R1/PTK2
5	**GO:0034599**	**cellular response to oxidative stress**	**ABCC1/CYP1B1/HIF1A/PARP1/MMP9/MMP2/EGFR/DAPK1**
6	GO:0043434	response to peptide hormone	CYP1B1/RAF1/GSK3B/PARP1/PRKACA/IGF1R/INSR/PIK3R1/PTK2
7	GO:0048511	rhythmic process	CYP1B1/GSK3B/PARP1/MMP2/TOP1/TOP2A/IGF1R/DRD4
8	GO:0007162	negative regulation of cell adhesion	CYP1B1/PLA2G2A/PLG/IL2/MMP2/SERPINE1/PIK3R1/PTK2
9	GO:0062197	cellular response to chemical stress	ABCC1/CYP1B1/HIF1A/PARP1/MMP9/MMP2/EGFR/DAPK1
10	GO:0009416	response to light stimulus	HIF1A/PARP1/MMP9/MMP2/KIT/EGFR/AURKB/PIK3R1
11	GO:1903008	cellular component disassembly	HIF1A/PLG/GSK3B/MMP9/MMP2/TOP2A/IGF1R/INSR/PIK3R1
12	GO:0097191	extrinsic apoptotic signaling pathway	RAF1/IL2/GSK3B/SERPINE1/TERT/PIK3R1/DAPK1
13	GO:0009411	response to UV	PARP1/MMP9/MMP2/EGFR/AURKB/PIK3R1
14	GO:1903036	positive regulation of response to wounding	PLG/SERPINE1/PLAU/IGF1R/PTK2
**15**	**GO:0006979**	**response to oxidative stress**	**ABCC1/CYP1B1/HIF1A/PARP1/MMP9/MMP2/EGFR/DAPK1**
16	GO:0051347	positive regulation of	KIT/EGFR/HSP90AA1/IGF1R/INSR/AURKB/DRD4/PTK2

		transferase activity	
17	GO:0008625	extrinsic apoptotic signaling pathway via death domain receptors	RAF1/GSK3B/SERPINE1/PIK3R1/DAPK1
18	GO:0009314	response to radiation	HIF1A/PARP1/MMP9/MMP2/KIT/EGFR/AURKB/PIK3R1
19	GO:0051897	positive regulation of phosphatidylinositol 3-kinase/protein kinase B signal transduction	KIT/EGFR/HSP90AA1/IGF1R/INSR/PTK2
20	GO:0043491	phosphatidylinositol 3-kinase/protein kinase B signal transduction	KIT/EGFR/HSP90AA1/IGF1R/INSR/PIK3R1/PTK2

Supplementary Table S3. Binding affinity of hydnocarpin on NOX2 and NOX4

Receptor	binding affinity (kcal/mol)
NOX2	-5.84 ~ -4.09
NOX4	-6.60 ~ -5.94

Reference

1. Bugnon, M.; Röhrig, U.F.; Goullieux, M.; Perez, Marta A.S.; Daina, A.; Michielin, O.; Zoete, V. SwissDock 2024: major enhancements for small-molecule docking with Attracting Cavities and AutoDock Vina. *Nucleic Acids Research* **2024**, *52*, W324-W332, doi:10.1093/nar/gkae300.
2. Grosdidier, A.; Zoete, V.; Michielin, O. SwissDock, a protein-small molecule docking web service based on EADock DSS. *Nucleic Acids Research* **2011**, *39*, W270-W277, doi:10.1093/nar/gkr366.
3. Eberhardt, J.; Santos-Martins, D.; Tillack, A.F.; Forli, S. AutoDock Vina 1.2.0: New Docking Methods, Expanded Force Field, and Python Bindings. *Journal of Chemical Information and Modeling* **2021**, *61*, 3891-3898, doi:10.1021/acs.jcim.1c00203.
4. Trott, O.; Olson, A.J. AutoDock Vina: Improving the speed and accuracy of docking with a new scoring function, efficient optimization, and multithreading. *Journal of Computational Chemistry* **2010**, *31*, 455-461, doi:https://doi.org/10.1002/jcc.21334.
